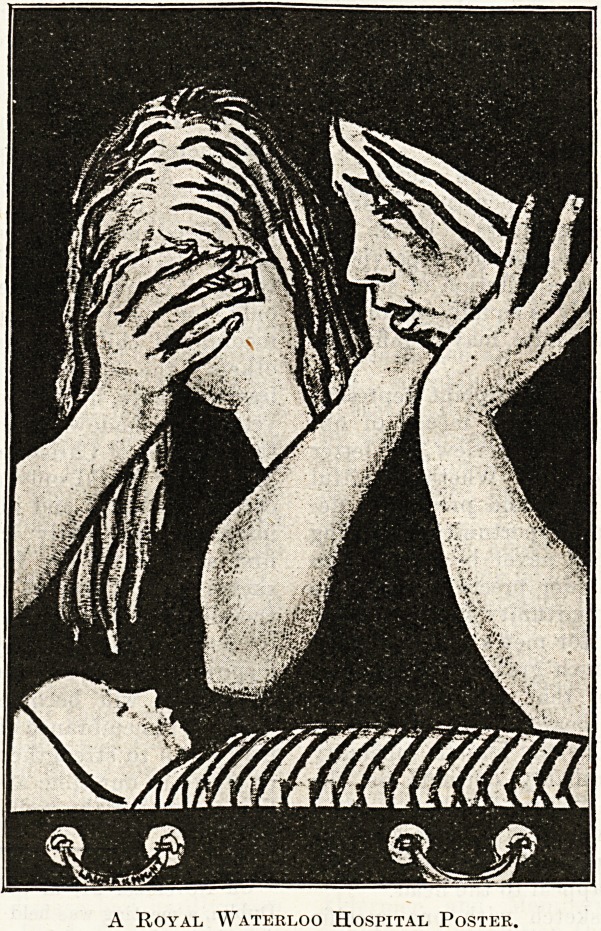# The Renewal of Vision in Hospital Administration

**Published:** 1922-01

**Authors:** Alexander Pym

**Affiliations:** Secretary of the Royal Waterloo Hospital for Children and Women


### THE RENEWAL OF VISION IN HOSPITAL ADMINISTRATION.

**By ALEXANDER PYM, Secretary of the Royal Waterloo Hospital for Children and Women.**

A RENEWAL of vision first and foremost is needed in the fierce anxieties which oppress hospital administration to-day. We know how easy it is in the distraction and pressure of routine to concentrate on points of detail to the exclusion of fundamental weakness. And above the eager discussion of rival schemes and remedies making themselves heard in all directions, no voice as yet has been raised with sufficient clearness to condemn that almost total lack of combination and undue conservatism which possibly more than anything else have invited bankruptcy.

A ROYAL WATERLOO HOSPITAL POSTER.

It is not unjust to say in general that hospitals comprise a number of independent units, self-sufficient and self-contained, pursuing a separate course, externally isolated and disunited. Each spins like a star in the charitable firmament, absorbed in shining, and happily oblivious of the radiance of others. Hospitals large and small, general and special those with medical schools and those without, each strike out a separate path, and the devil take the hindmost!

Even internally a lack of co-ordination appears. In a crisis, tradition forbids that any should speak with authority. The more urgent the matter, the more imperative the demand for reference, for consultation, before decision. The layman and expert, the professional and voluntary helper, auxiliary organisations and the Board, create in themselves a diversity of interest which insists on recognition.

Competition, it will be urged, is encouraged and efficiency ensured by such a system. But there is another and less pleasant side. A number of dogs fighting for a bone represents something more than competition. Conditions, too, of work, locality and circumstance vary, no doubt, enormously between hospital and hospital. But it may be wondered if the difficulties, as such, are really greater or more insuperable than those of other large organisations; and whether competition, individuality or anything else can sufficiently compensate for the undeniable loss of dynamic power which such a system entails.

The appointment of numerous hospital committees and organisations at first sight might be taken to refute the charge. What, it might be asked, of the Metropolitan Funds, which systematise and control methods of finance? The British Hospitals Association, with its Regional Committees, or, more recently, as a result of Lord Cave's report, the Voluntary Hospitals Commission? to mention only a few. But does not the very multiplicity of such bodies tend to confirm rather than dispose of the danger? Liable to an unwieldiness of composition, their functions inadequately defined and prone to overlap, none can be accepted as supreme, none absolutely representative. And the authority of each is in some degree confused and challenged by the existence of the others.

It has become difficult, in fact, to see the wood for the trees; and a point has been reached at which no methods, however ingenious, can hope to succeed unless they involve a return to first principles. Before matters of detail, vision first must be renewed and purpose strengthened. To whatever extent mechanical routine obscures the fact, there is one thing, and one thing only, which makes effort worth while. It is the thread which binds the highest to the lowest, the scrubber to the Royal fellow-worker. We have a common foe; a common and splendid cause. It lies in our power to hustle and break the enemy, if only we will close our ranks and present a united front. In these days, what is wanted more than anything is —Brotherhood.

We will try, then, to regain the spirit of the Crusader. And from this standpoint if we proceed to consider the second source of weakness we shall probably confess that an uninspired adherence to old methods, a lack of vision, is to be held responsible, at least in part, for present difficulties. For no business in the country has more attractive wares to offer than a hospital. No subject, if rightly treated, has a greater power to grip imagination. How comes it, then, that while commerce attaches increasing

**The Renewal of Vision in Hospital Administration—** *(cont.).*

importance to atmosphere and publicity, hospitals, except for the almost automatic issue of desperate appeals, remain comparatively dumb ? What about their wonderful history and achievements ? Their romance and mystery ? Their silent but unquestioned influence from birth to the grave on every being in these islands ?

If it is indeed true, as often stated, that in the reaction following war emotions we have become temporarily relaxed and numb, old cries must prove more than ever inadequate. It can no longer suffice through the medium of post and press to issue by the thousand, as from a slot-machine, the same type of letter that did duty ten years ago. The generosity of the public has not changed. It is still ready to give if the right chord is struck. But it is frankly tired of the stock letter, and more than tired of the professional whine.

Our lack of enterprise, however, has excuse. In the background lurks the fear of incurring criticism on the ground of high expenditure ; herein lies possibly the one defect of close financial supervision. For money is not the only true criterion of success. To create the right spirit surely is equally important. A kindly thought, or a widow's mite, may be infinitely more valuable.

Appeals, in fact, from this standpoint represent something more than a stereotyped means of obtaining money. Whether by interview or letter each appeal is definitely creative. Whether fruitful in result or not, each originates and presents a definite chance of self-denial, an opportunity of helping another—a chance which can never be repeated in exactly the same way or under precisely the same conditions. It is both an opportunity and a privilege. Add to this that it is good for men's souls to give ; if the giving be hard, so much greater the value of the gift. It follows, then, that money extracted from another's pocket can bear no real likeness to the rabbit from a hat. Though human ingenuity varies, less importance probably depends on the form—the ingenuity of the " stunt "—than is often supposed. A right spirit will inspire fresh methods and renew the old. An appeal to the heart is incomparably better than an appeal to the head.

To complete this brief sketch with one specific instance :—

More than a century has passed since the Royal Waterloo Hospital for Children and Women was first founded as a children's dispensary. Its position to-day is neither better nor worse than that of its neighbours. The passing of time, as in other cases, has brought with it enormous development ; few who know the sketch of the small establishment of 1816 at St. Andrew's Hall could hope to recognise its modern counterpart in the Waterloo Road. Here, as elsewhere, the unexampled depression of the last twelve months, accompanied—and in this case largely accounted for—by the burden of a long-needed extension, produced a crisis ; a similar disappointment of hopes. And hope had been unusually high, for the year 1918 closed with the happiest prospects.

But the sudden change from comparative affluence to the verge of bankruptcy taught at least one useful lesson. To obtain money charities need not plead beggary. Help never proved more spontaneous or general than when the hospital could proclaim, as, indeed, it did, a balance in hand. Interest was stimulated, and friends showed approval by giving. Rank heresy, no doubt, to preach prosperity in hospital finance ! Yet the experience remains a valuable one.

This institution, too, has escaped no more than its neighbours that tendency to isolation which has already been noted as a failing of philanthropic work. A few months ago, however, a chance occurred to do something to lessen the reproach, and eleven societies and organisations interested particularly in children decided to combine forces in a Christmas Carnival and Children's Fair, to be given at the Albert Hall from December 26 to January 4 inclusive.

The value of such an enterprise lies deeper than that merely of a demonstration, or a money-making device. An endeavour primarily to provide entertainment for children of all ages, it also bears witness to the friendliness and solidarity of interest which ultimately unites all societies. During the period of ten days in question every facility will be given, not only for individual enjoyment, but through the medium of children's parties, for the enjoyment of others ; the entire proceeds being devoted to those less fortunate little ones on whose Christmas this year the shadows are already darkening. With this thought of Christmas we may fittingly conclude. The writer would only ask pardon for leading from certain problems and ideals in general to particular instances apparently irrelevant ; it may seem at first sight a far cry from the main theme of this sketch to a Carnival. But the distance we have travelled is not really so great. For where can vision be renewed more clearly, or incentive more readily inspired, than in the glances of children ? And the connection will become clearer in the months to follow if remembrance of glad scenes and bright faces may serve to strengthen a purpose too often feeble—to strive in our time and generation, as far as in us lies, to further the well-being of our fellow-men.

---

On November 24, in the Theatre of the Royal Society, Dublin, a meeting was held by the Women's National Health Association for organising a conference on child-welfare work. The meeting was attended by representatives of organisations interested in child-welfare work, and it is hoped that the movement may be instrumental in reducing the high infant mortality rate that prevails in Dublin and other Irish towns.

---

Six additional scholarships for members of the College of Nursing will be competed for at an examination to be held in January. The donors are : (1) Miss Barton, R.R.C.—£150 for a Sister-Tutor Scholarship for a member trained in a Poor-law Infirmary ; (2) Messrs. Joseph Nathan & Co. (Glaxo Department)—two scholarships of £50 each, to enable the holders to obtain the certificate of the Central Midwives Board ; and (3) Messrs. Cadbury—two scholarships of £150 each, to be awarded in 1922 and 1923 respectively, to enable the holders to take the special qualification of one year's training for Sister-Tutors at King's College for Women, University of London.

## Figures and Tables

**Figure f1:**